# Serum resistin is predictive marker of development of new digital ulcers in systemic sclerosis

**DOI:** 10.1007/s10238-021-00756-2

**Published:** 2021-08-30

**Authors:** Chiara Pellicano, Giorgia Leodori, Amalia Colalillo, Luca Navarini, Antonietta Gigante, Edoardo Rosato

**Affiliations:** 1 Department of Translational and Precision Medicine, Lazio, Sapienza University of Rome, Viale dell’Università 37, 00185 Rome, Italy; 2 Unit of Allergology, Clinical Immunology and Rheumatology, Department of Medicine, Campus Bio-Medico University, Rome, Italy

**Keywords:** Resistin, Digital ulcers, Systemic sclerosis, Angiogenesis

## Abstract

Systemic sclerosis (SSc) is autoimmune disease characterized by endothelial dysfunction and microvascular damage. Resistin has been implied in microvascular dysfunction. Objective of this study is to evaluate the association between baseline resistin and development of new digital ulcers (DUs) in SSc patients. At baseline, serum resistin has been assessed in 70 female SSc patients and 26 healthy controls (HC). In SSc patients, clinical assessment was performed at baseline and after a 52-weeks follow-up. Serum resistin level was increased in SSc patients compared to HC [5.89 ng/ml (2.5 ng/ml–8.1 ng/ml) vs 2.3 ng/ml (0.4 ng/ml–2.4 ng/ml), *p* = 0.0004)]. Resistin was lower (*p* = 0.005) in SSc patients with early capillaroscopic pattern than patients with active or late capillaroscopic pattern [2.49 ng/ml (0.89 ng/ml–5.81 ng/ml) vs 7.11 ng/ml (3.48 ng/ml–11.35 ng/ml) and 6.49 ng/ml (3.35 ng/ml–8.87 ng/ml), respectively]. After a 52-weeks follow-up, 34 (48.6%) patients developed new DUs. Median serum resistin was significantly higher in patients with new DUs than in patients without new DUs [6.54 ng/ml (3.35 ng/ml–11.02 ng/ml) vs 4.78 ng/ml (1.06 ng/ml–7.6 ng/ml), *p* = 0.019]. Kaplan–Meier curves show a significantly reduced free survival from DUs in patients with increased resistin (*p* = 0.002). In multivariate analysis, resistin is associated with the development of new DUs. Increased serum resistin level is a predictive marker of new DUs in SSc.

## Introduction

Systemic sclerosis (SSc) is an autoimmune disease characterized by microvascular damage, autoimmunity-mediated inflammation and fibroblast activation. In SSc, the endothelial dysfunction is one of the most important features that involves both the macro- and microvascular network [1]. Digital ulcers (DUs) are the main vascular complication of chronic hypoxia consequent to endothelial dysfunction [2]. High modified Rodnan skin score (mRSS) and diffuse cutaneous (dcSSc) subset, anti-topoisomerase I antibody, higher value of systolic pulmonary arterial pressure (sPAP), increased intrarenal arterial stiffness, late capillaroscopic pattern and increased percentage of CD21^low^ B cells are known risk factors for development of new DUs in SSc patients [3–8].

Adipose tissue is involved in many inflammatory processes. Various soluble factors produced by adipose tissue, known as adipocytokines or adipokines, have been characterized, such as resistin, adiponectin, leptin [9, 10].

Human resistin is a 12.5 kDa polypeptide, which belongs to a family of cysteine-rich proteins, called resistin-like molecules (RELMs), implicated in the regulation of inflammatory processes [11]. Resistin has a specific role in the development of microvascular damage and endothelial dysfunction [12, 13]. When incubated with resistin, endothelial cells (EC) respond by a greater production of endothelin-1 (ET-1) and augment ET-1 mRNA expression [14]. Moreover, although resistin did not affect the basal release of endothelium-derived nitric oxide (NO), it significantly augmented pathological inflammation increasing the expression of the vascular cell adhesion molecule 1 (VCAM-1) and the chemoattractant chemokine MCP-1, involved in early atherosclerotic lesion formation [15].

Several studies indicate that resistin is associated with specific organ manifestations in SSc, especially the vascular ones [16–20]. Sawicka et al. [18] found an association between serum resistin and interstitial lung disease (ILD), arthralgia, esophageal involvement and inflammatory parameters in SSc patients. Masui et al. [19] hypothesized a possible role of serum resistin level as marker for pulmonary vascular involvement in SSc, suggesting a possible contribution of resistin to the pathogenesis of SSc pulmonary arterial hypertension (PAH). Moreover, serum resistin level is elevated in SSc patients with DUs compared to SSc patients without DUs [20].

Aim of this study is to evaluate the role of serum resistin level in development of new DUs in SSc patients.

## Material and methods

### Subjects

Seventy consecutive SSc females patients [median age 55 years (IQR 43 years–62 years)], fulfilling the American College of Rheumatology/European League Against Rheumatism Collaborative Criteria for SSc, were enrolled in this study [21]. Forty-one (58.6%) had dcSSc and twenty-nine (41.4%) had limited cutaneous SSc (lcSSc) according to Le Roy et al. [22]. Demographic and clinical features of SSc patients are shown in Table 1.

**Table 1:** Demographic and clinical characteristics of SSc patients and HC

	SSc	HC	*p*
Female, *n* (%)	70 (100)	26 (100)	1.0
Age, years, median and IQR	55 (43–62)	50 (37–64)	0.09
Disease duration, years, median and IQR	12 (9–17)		
dcSSc, *n* (%)	41 (58.6)	–	–
Nailfold capillaroscopic pattern			–
Early, *n* (%)	17 (24.3)	–	
Active, *n* (%)	24 (34.3)	–	
Late, *n* (%)	29 (41.4)	–	
SSc-specific autoantibodies			–
Anti-topoisomerase I, *n* (%)	44 (62.9)		
Anti-centromere, *n* (%)	23 (32.9)		
None, *n* (%)	3 (4.3)		
mRSS, median and IQR	11 (6–18)	–	–
DAI, median and IQR	2 (1–5)	–	–
DSS, median and IQR	4 (3–7)	–	–
New digital ulcers, *n* (%)	34 (48.6)	–	–
BMI, Kg/m^2^, median and IQR	22.7 (19.4–26)	23.2 (19.5–26.9)	0.5
Systemic arterial hypertension, n (%)	18 (26)	2 (8)	0.05
Resistin, ng/ml, median and IQR	5.89 (2.49–8.09)	2.3 (0.4–2.4)	**0.0004**

The median value of resistin (ng/ml) is significantly higher in SSc patients than healthy controls (p=0.0004)

*SSc* systemic sclerosis, *HC* healthy controls, *IQR* interquartile range, *dcSSc* diffuse cutaneous systemic sclerosis, *mRSS* modified Rodnan skin score, *DAI* disease activity index, *DSS* disease severity scale, *BMI* body mass index

Exclusion criteria were active malignancies, acute and chronic kidney disease, recent surgery (< 1 year), recent cardiovascular or cerebrovascular events (< 1 year), recent exposition to chemotherapy or implantation of autologous adipose tissue-derived cells for the treatment of DUs, peripheral arterial disease, coagulopathies, diabetes. Smokers, pregnancy and breastfeeding were excluded.

Twenty-six healthy controls (HC) matched for sex and age [median age 50 years (IQR 37 years–64 years)] were also enrolled in this study.

The subjects’ written consent was obtained according to the Declaration of Helsinki, and the study was approved by the ethics committee of Sapienza University (IRB approval 377).

### Clinical assessment

The main clinical indexes were assessed for all SSc patients at baseline and every six months for a follow-up period of 52 weeks. Skin involvement was assessed by mRSS [23]. The activity and severity of disease were assessed by disease activity index (DAI) [24] and disease severity scale (DSS) [25], respectively. Nailfold videocapillaroscopy (NVC) was performed with a videocapillaroscope (Pinnacle Studio Version 8) equipped with a 500 × optical probe. The nailfold of the second, third and fourth finger was examined in each patient. According to Cutolo et al., patterns identified include early, active and late [26]. DUs were defined according to Amanzi et al. [27].

### Serum resistin quantification

Serum resistin level was determined in SSc patients by commercial ELISA kit (Human Resistin Quantikine ELISA Kit, R&D Systems, USA), according to the instructions provided by the manufacturer with an assay range of 4.1 ng/ml–12.1 ng/ml.

### Statistical analysis

SPSS version 25.0 software was used for statistical analysis. Shapiro–Wilk test was used to evaluate normal distribution of data. All results are expressed as median and interquartile range (IQR). Group comparisons were made by Mann–Whitney U test. Spearman’s rank correlation coefficient was used to test for associations between numerical variables. The chi-square test or Fisher’s exact test was used to compare categorical variables. All time-to-event endpoints were estimated by the Kaplan–Meier method and analyzed by the logrank test. Hazard ratios with 95% confidence intervals were calculated by use of Cox regression models. *p* values < 0.05 were considered significant.

## Results

Table 1 shows demographic and clinical characteristics of SSc patients and HC. Median disease duration was 12 years (9 years–17 years). NVC pattern was early in 17 (24.3%), active in 24 (34.3%) and late in 29 (41.4%) SSc patients. Forty-four (62.9%) patients were positive for anti-topoisomerase I autoantibodies and 23 (32.9%) had anti-centromere antibodies. Median values of mRSS, DAI and DSS were 11 (IQR 6–18), 2 (IQR 1–5) and 4 (IQR 3–7), respectively.

Median serum resistin level was significantly higher in SSc patients compared to HC [5.89 ng/ml (2.5 ng/ml–8.1 ng/ml) vs 2.3 ng/ml (0.4 ng/ml–2.4 ng/ml), *p* = 0.0004]. Resistin was lower (*p* = 0.005) in SSc patients with early capillaroscopic pattern than patients with active or late capillaroscopic pattern [2.49 ng/ml (0.89 ng/ml5.81 ng/ml) vs 7.11 ng/ml (3.48 ng/ml–11.35 ng/ml) and 6.49 ng/ml (3.35 ng/ml–8.87 ng/ml), respectively]. SSc patients with new DUs had significantly higher median serum resistin level compared to SSc patients without new DUs [6.54 ng/ml (3.35 ng/ml–11.02 ng/ml) vs 4.78 ng/ml (1.06 ng/ml–7.6 ng/ml), *p* = 0.019]. Resistin level did not significantly differ according to SSc subset or SSc-specific autoantibodies. All data are summarized in Table 2. We did not find any correlation between resistin serum level and age, duration of disease, mRSS, DAI, DSS and BMI.

**Table 2:** Disease variables and resistin serum level in SSc patients

	Resistin serum level, ng/ml, median and IQR	*p*
SSc Subset		
dcSSc	6.32 (1.94–7.78)	0.793
lcSSc	5.81 (3.28–8.09)	
Nailfold capillaroscopic pattern		
Early	2.49 (0.89–5.81)	**0.005**
Active	7.11 (3.48–11.35)	
Late	6.49 (3.35–8.87)	
SSc-specific autoantibodies		
Anti-topoisomerase I	6.04 (1.74–9.27)	0.98
Anti-centromere	5.81 (3.28–7.99)	
None	7.58 (0.73–8.68)	
New digital ulcers		
Yes	6.54 (3.35–11.02)	**0.019**
No	4.78 (1.06–7.6)	

The median value of resistin (ng/ml) is higher (p=005) in active and late capillaroscopic patterns than early capillaroscopic pattern.

*SSc* systemic sclerosis, *IQR* interquartile range, *dcSSc* diffuse cutaneous systemic sclerosis, *lcSSc* limited cutaneous systemic sclerosis

**Fig. 1:**
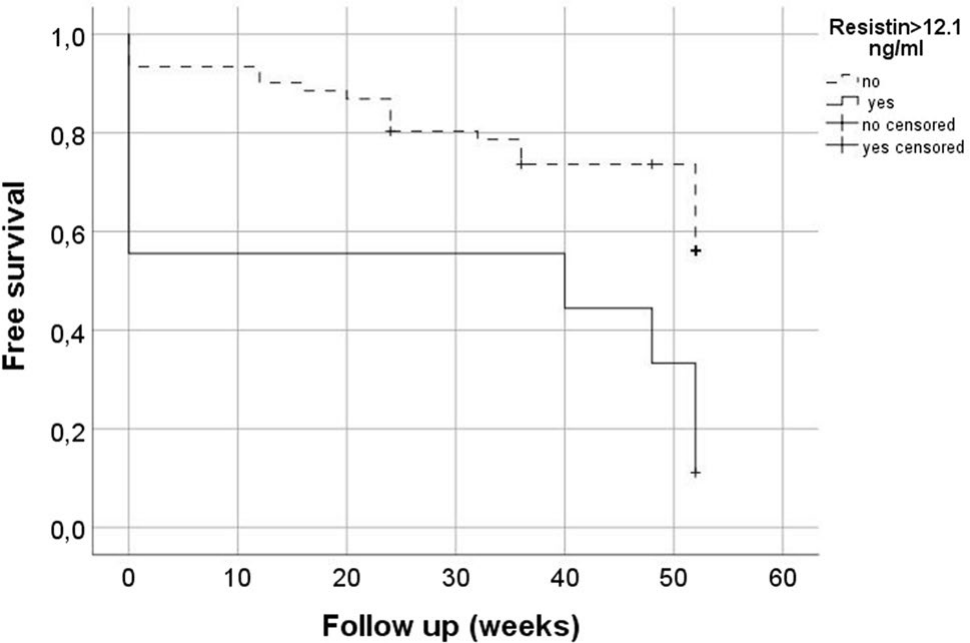
Kaplan–Meier curves for the onset of new digital ulcers during a 52 weeks follow-up period in systemic sclerosis patients with normal or increased serum resistin level. Systemic sclerosis patients with resistin serum level > 12.1 ng/ml (solid line) have a significantly reduced free survival from digital ulcers development than patients with normal resistin level (dashed line)

After a 52-weeks follow-up period, 34 (48.6%) SSc patients developed new DUs. Kaplan–Meier analysis comparing new DUs free survival in SSc patients with increase in resistin serum level demonstrated that those with resistin serum level > 12.1 ng/ml develop new DUs (LogRank = 0.002) (Fig. 1).

In univariate regression analysis, resistin [HR 1.094 (95% CI 1.035–1.157); *p* = 0.001], early capillaroscopic pattern [HR 0.205 (95% CI 0.063–0.674); *p* = 0.009], mRSS [HR 1.077 (95% CI 1.022–1.134); *p* = 0.006], DAI [HR 1.215 (95% CI 1.079–1.369); *p* = 0.001] and DSS [HR 1.152 (95% CI 1.058–1.253); *p* = 0.001] are predictive markers of new DUs development (Table 3).

**Table 3:** Univariate and multivariate analysis with hazard ratio (HR) and confidence interval (CI) for the development of new digital ulcers in SSc patients with increased resistin level

	Univariate analysis		Multivariate analysis	
	HR (CI)	*p*	HR (CI)	*p*
Resistin	1.094 (1.035–1.157)	**0.001**	1.078 (1.014–1.145)	**0.016**
Nailfold capillaroscopic pattern (early)	0.205 (0.063–0.674)	**0.009**	0.381 (0.108–1.345)	0.134
SSc subset	1.915 (0.914–4.010)	0.085	–	–
SSc-specific autoantibodies	0.495 (0.224–1.096)	0.222	–	–
Disease duration	0.996 (0.936–1.061)	0.905	–	–
mRSS	1.077 (1.022–1.134)	**0.006**	1.011 (0.933–1.095)	0.789
DAI	1.215 (1.079–1.369)	**0.001**	1.070 (0.807–1.420)	0.637
DSS	1.152 (1.058–1.253)	**0.001**	1.074 (0.872–1.323)	0.502

The median value of resistin (ng/ml) is higher (p=0019) in SSc patients with new digital ulcers than in SSc patients without new digital ulcers

*SSc* systemic sclerosis, *mRSS* modified Rodnan skin score, *DAI* disease activity index, *DSS* disease severity scale

In multivariate analysis, only resistin is a predictive marker of new DUs development [HR 1.078 (95% CI 1.014–1.145); *p* = 0.016] (Table 3).

## Discussion

In this study, we found increased serum resistin level in SSc patients compared to HC. We found that serum resistin is increased in SSc patients with early NVC pattern and in patients with new DUs.

The association of serum resistin with DUs in SSc was described in cross-sectional studies [19, 20]. It is well known that ET-1 is strongly upregulated in SSc patients and it plays a key role in the pathogenesis of major vascular complications of SSc such as DUs and PAH [28, 29]. In RAPIDS 1–2, authors demonstrated that ET-1 is increased in SSc patients with new DUs and bosentan, a dual endothelin receptor antagonist, treatment was associated with a 30% reduction in the number of new DUs. The authors conclude that treatment with bosentan may be effective in preventing new DUs [30, 31]. Olewicz-Gawlik et al. [20] demonstrated that SSc patients with DUs have higher serum resistin level compared to SSc patients without DUs. Few studies demonstrate that resistin is increased in PAH. Masui et al. [19] found serum resistin levels significantly increased in patients with elevated right ventricular systolic pressure (RVSP) than in those with normal RVSP; the authors concluded that elevation of resistin levels is associated with proliferative obliterative vasculopathy, especially pulmonary arterial involvement leading to PAH. In the same study, the authors found an higher prevalence of DUs in SSc patients with elevated serum resistin levels supporting the previous data regarding the pro-angiogenic property of resistin [14, 15]. A higher serum resistin level seems to be associated with ILD, arthralgia, esophageal involvement and inflammatory parameters in SSc patients [18]. We can suppose that through the increased production of ET-1, resistin may determine a proliferative vascular disease, characterized by a mio-intimal proliferation, leading to the typical blood flow alterations in SSc [16, 32, 33]. We hypothesized that resistin may play a role in the pathogenesis of new DUs by up-regulation of endothelial ET-1 synthesis. In SSc patients, high serum resistin level was associated with non-vascular SSc complications.

In this study, after a 52-weeks follow-up, 48.6% SSc patients developed new DUs and median serum resistin level was significantly higher in patients with new DUs than in patients without new DUs. In univariate analysis, we demonstrated that resistin, early capillaroscopic pattern, mRSS, DAI and DSS are predictive markers of new DUs, conversely, in multivariate analysis, only resistin is a predictive marker of new DUs.

In literature, markers of development of new DUs are widely reported: mRSS, dcSSc, anti-topoisomerase I antibody, late capillaroscopic pattern, PAH, increased intrarenal arterial stiffness and increased percentage of CD21^low^ B cells [3–8]. Hachulla et al. [3] found that development of new DUs is associated with high mRSS and development of DUs typically occurred within 5 years of the first non-Raynaud clinical symptom of SSc in the majority of patients. In addition, Tiev et al. [4] demonstrated that early onset of SSc, increased duration of SSc, high mRSS, and presence of anti-topoisomerase I antibodies were associated with prior or current DUs. DUs development correlates with capillaroscopic damage and Sebastiani et al. [5] proposed CSURI as a novel tool with the ability to predict the development of DUs in SSc patients. In a recent EUSTAR observational cohort study of patients with newly diagnosed DUs, dcSSc, anti-topoisomerase I antibody and PAH were associated with presence of any DU at the prospective visit [6]. Rosato et al. [7] demonstrated that renal resistive index is a predictive marker of development of new DUs: It is increased in SSc patients with new DUs compared to patients without new DUs and the subclinical renal vasculopathy correlates to digital vascular damage. The authors concluded that Doppler indices of intrarenal stiffness are reliable markers of new DUs occurrence and could be used in association with the capillaroscopic and clinical findings or serologic tests for the identification of patients at high risk of developing DUs [7].

Moreover, Visentini et al. [8] found that the median percentage of CD21^low^ B cells was significantly higher in patients with new DUs than in patients without new DUs and showed a significantly reduced free survival from new DUs in SSc patients with CD21^low^ B cells > 10%. In multivariate analysis, CD21^low^ B cells were associated with the development of new DUs and the authors concluded that CD21^low^ B cells > 10% are a predictive marker of onset of new DUs [8].

For the first time, we demonstrated that that serum resistin level is a predictive marker of development of new DUs in SSc patients.

We can conclude that serum resistin level may be a predictive marker of new DUs development in SSc patients. Larger prospective studies are needed to evaluate the potential role of serum resistin as a biomarker predictive of new DUs.

## Data Availability

All data are presented in the main manuscript.
